# Expanding the Biocatalytic Scope of Enzyme-Loaded Polymeric Hydrogels

**DOI:** 10.3390/gels7040194

**Published:** 2021-11-03

**Authors:** Zhongbiao Tan, Muhammad Bilal, Ali Raza, Jiandong Cui, Syed Salman Ashraf, Hafiz M. N. Iqbal

**Affiliations:** 1 School of Life Science and Food Engineering, Huaiyin Institute of Technology, Huaian 223003, China; tanzb@hyit.edu.cn; 2 School of Biomedical Engineering, Shanghai Jiao Tong University, 800 Dongchuan Road, Shanghai 200240, China; aliraza86@sjtu.edu.cn; 3 State Key Laboratory of Food Nutrition and Safety, Laboratory of Industrial Fermentation Microbiology, Ministry of Education, Tianjin University of Science and Technology, No 29, 13th, Avenue, Tianjin Economic and Technological Development Area (TEDA), Tianjin 300457, China; jdcui@tust.edu.cn; 4 Department of Biology, College of Arts and Sciences, Khalifa University, Abu Dhabi P.O. Box 127788, United Arab Emirates; syed.ashraf@ku.ac.ae; 5 Center for Biotechnology (BTC), Khalifa University of Science and Technology, Abu Dhabi P.O. Box 127788, United Arab Emirates; 6 Tecnologico de Monterrey, School of Engineering and Sciences, Monterrey 64849, Mexico

**Keywords:** polymeric gels, bio-catalysis, multi-functional characteristics, environmental engineering

## Abstract

In recent years, polymeric hydrogels have appeared promising matrices for enzyme immobilization to design, signify and expand bio-catalysis engineering. Therefore, the development and deployment of polymeric supports in the form of hydrogels and other robust geometries are continuously growing to green the twenty-first-century bio-catalysis. Furthermore, adequately fabricated polymeric hydrogel materials offer numerous advantages that shield pristine enzymes from denaturation under harsh reaction environments. For instance, cross-linking modulation of hydrogels, distinct rheological behavior, tunable surface entities along with elasticity and mesh size, larger surface-volume area, and hydrogels’ mechanical cushioning attributes are of supreme interest makes them the ideal candidate for enzyme immobilization. Furthermore, suitable coordination of polymeric hydrogels with requisite enzyme fraction enables pronounced loading, elevated biocatalytic activity, and exceptional stability. Additionally, the unique catalytic harmony of enzyme-loaded polymeric hydrogels offers numerous applications, such as hydrogels as immobilization matrix, bio-catalysis, sensing, detection and monitoring, tissue engineering, wound healing, and drug delivery applications. In this review, we spotlight the applied perspective of enzyme-loaded polymeric hydrogels with recent and relevant examples. The work also signifies the combined use of multienzyme systems and the future directions that should be attempted in this field.

## 1. Introduction

Hydrogels are solid-like and viscoelastic but deformable biomaterials that contain a substantially diluted network in aqueous media [1]. The network is composed of physically or chemically cross-linked hydrophilic polymer, which is responsible for structural steadiness and circumvents the polymer dissolution into the aqueous medium. Preserving their shapes while the absorption of a huge amount of water constitutes hydrogels, the promising candidates to encapsulate various proteins, drugs, and other biological molecules [2,3]. Moreover, a 3-dimensional (3D) network exhibits a considerable permeability for metabolites, oxygen, small molecules, and other water-soluble constituents. All these desired properties and viscoelastic traits are likely to render hydrogels as good carrier materials for biomolecules and mimic the mucus or extracellular matrix [4,5]. Wichterle and Lim synthesized the first hydrogel (hydrophilic gel) in 1960 with an ambitious objective to create a “plastic” in long-lasting contact with human tissues. Since then, the demands, variety, applications prospects of hydrogels have expanded immensely in diverse arenas of biotechnology, such as molecular diagnostics, tissue engineering, wound healing, and drug delivery [6,7,8].

Hydrogels have been generated from a large variety of natural polymers, such as alginate, dextran, chitosan, and hyaluronic acids, as well as synthetic polymers, including poly(2-hydroxyethyl methacrylate) (PHEMA), poly(N-isopropylacrylamide) (PNIPAAm), and polyethylene glycol (PEG). Due to their hydration properties, these polymeric materials are resilient to cell adhesion and protein adsorption [9]. The specific attributes of hydrogels are originated from the repeating molecular units of the polymeric chains. These units can be further chemically modified for providing networks with adjustable or additional functional properties. The presence of functional groups influences the properties of the hydrogel, such as the processability, stiffness, and swelling ratio. Based on chemical modification, hydrogels are categorized as degradable, charged, pH-responsive, thermo-responsive, self-healing, magnetic, or swell upon stimuli. The creation of solution-like microenvironment by hydrogel matrix protects and stabilizes the structural organization of delicate biomolecules that may otherwise denature on exposure to surfaces [10]. 

## 2. Hydrogels as Immobilization Matrix

The three-dimensional porous architecture of hydrogels increases the material’s surface area, facilitating the immobilization of a large amount biomolecules, cells, proteins, and enzymes. The prevention or reduction of non-specific attachment is one of the desired features of hydrogels for utilizing a robust immobilization support matrix. This is presumably ascribed to the polymers antifouling properties and the solution-like microenvironment generated by the hydrogel. Consequently, biomolecules, which are not chemically addressed will be unlikely to attach to the surface. Preservation of the native structure of biomolecules and enzymes is a critical prerequisite for sensitivity, feasibility, and specificity in numerous bio-applications. Updike and Hicks devised one of the first systems in 1967 that implicated the entrapment of biomolecules [11]. They carried out the entrapment of glucose-oxidase in a polyacrylamide gel to increase its functional steadiness and streamlining the biosensor development [12]. Various strategies have been envisioned to encapsulate biomolecules. Higher loading efficiency, better approachability, and the creation of a more biocompatible microenvironment by the flexible hydrogels are notable merits of encapsulation over direct immobilization. A plethora of investigations has been reported for encapsulating biomolecules and enzymes into hydrogel networks. These studies found that encapsulation results in the preservation of the native conformation of the protein and thus improves enzyme bioactivity [13]. Adsorption, entrapment, encapsulation, covalent linking, and cross-linking are the main methodologies for enzyme immobilization. By the adsorption method, the enzyme is adsorbed to the support through weak forces, such as van der Waals forces, hydrogen bond, and acid-base bond, which contribute to a high risk of enzyme leaching from the matrix. By means of entrapment and encapsulation, the enzyme can be physically restricted and concentrated in the matrix. Covalent bonds in covalent linking and cross-linking methods make robust interactions between enzyme and matrix, which lower the risk of enzyme leakage. To avoid the risk of enzyme leakage and acquire better enzymatic action, a combination of different methods, i.e., entrapment and covalent linking, have been proposed to realize a robust immobilization [13].

## 3. Hydrogels for Biocatalysis

Enzyme-loaded nanocomposites and hydrogels are widely used in biocatalytic industrial processes. Horn et al. [14] developed a new polymer-based hydrogels that contained itaconic acid (ITA), 2-hydroxyethyl methacrylate (HEMA), *N*,*N*’-diethyl-1,3-bis(acrylamido)propane (BAAP) and 2-2-(ethoxycarbonyl)prop-2-en-1-yl-oxy-ethyl phosphonic acid (ECPPA) as the cross-linking agents. The as-synthesized hydrogel was applied to immobilize laccase from *Trametes versicolor* for enhancing its catalytic properties. ATR-FTIR spectroscopy evidenced that the covalent attachments of laccase led to improved enzymatic activity. The enzyme-incorporated hydrogels were employed as granules and coatings on porous Al_2_O_3_ ceramic substrates for the bioconversion of organic pollutants such as diclofenac, bisphenol A, triclosan, 17α-ethinylestradiol, paracetamol, *p*-chlorophenol, and 4-tert-octylphenol. After 24 h of enzyme-immobilized hydrogels exposure, the maximum bioconversion of triclosan (>90%) was achieved in water, whereas bisphenol A, *p*-chlorophenol and 17α-ethinylestradiol reached a bioconversion between 60% and 70%. 

Natural polysaccharide-derived hydrogels are exceptional candidates for a large number of medical, pharmaceutical, and biological applications owing to good biodegradability, biocompatibility, and amenability to chemical modification. The presence of multiple bioactive amino, carboxylic and hydroxyl groups in polysaccharide bio-macromolecules are likely to be easily functionalized and crosslinked by a variety of chemicals [15]. Chitosan is a widely studied biopolymer in recent years for immobilizing enzymes and biological molecules due to its inexpensive, biocompatibility, non-toxicity, and good mass transfer properties [16]. It is a linear copolymer of D-glucosamine and *N*-acetyl-d-glucosamine formed by chitin N-deacetylation in variable degrees. Chitin is a precursor for chitosan and is mainly produced from crustaceans. It is regarded as the second most plentiful naturally occurring polysaccharide on earth. Many studies have documented the preparation of chitosan-based hydrogels by simple gelation methods in sodium hydroxide solution for the immobilization of various kinds of enzymes. However, such hydrogels exhibit limited mechanical stability and thermal tolerance, thus rendering it impractical for large-scale applications [17]. To overcome these drawbacks, researchers have suggested the use of blending synthetic and natural polymers to increase the mechanical properties and thermophysical of chitosan hydrogels [18]. On the other hand, polymer amalgamation is, expensive, time intensive, demands technical expertise, and reduces the adsorptive capability of enzymes.

Recently, the utilization of anionic surfactants has gained interest as gelling solutions to generate chitosan hydrogels with improved stability and mechanical strength. Dwamena et al. [19] developed a novel technique to immobilize *Thermoanaerobacter ethanolicus* alcohol dehydrogenase (TeSADH) enzyme on porous chitosan hydrogel capsules, which were prepared by coupling NaOH and sodium dodecyl sulfate as gelation solution. This novel immobilization method resulted in enhanced mechanical strength of the hydrogel beads and improved the thermal stability and binding ability of enzyme. In contrast to free enzyme, the immobilized biocatalytic system presented 68% improvement in producing [R]-1-phenylethanol. After 2 h incubation at 80 °C, it maintained about 25% of its original catalytic activity, while the soluble enzyme is denatured and lost its entire activity at this temperature.

Chitosan and Arabic gum-based hydrogels are robust porous supports for immobilizing enzymes. The catalytic stability of β-d-galactosidase upon immobilization on chitosan and Arabic gum-based hydrogels was inspected to catalyze lactose breakdown. Results revealed freeze-dried hydrogels as more promising for immobilizing β-d-galactosidase compared to air-dried hydrogels. Arabic gum-based hydrogel, chitosan hydrogel network showed superior immobilization capacity for β-d-galactosidase. After immobilization in both types of hydrogel network, β-d-galactosidase was capable of reusing in five successive runs to catalyze lactose hydrolysis. Both types of developed biocatalysts were highly stable in acidic environments compared to free counterparts, which is a fascinating benefit to applying immobilized β-d-galactosidase in various applications [20].

Pectin is extensively produced from the cell walls of an agro-industrial residue, citrus peels. In this polysaccharide, galacturonic acid units are linked through α-[1 → 4] bonding [21]. Pectin has been widely applied as a support matrix for immobilizing various enzymes and bioactive molecules, such as ConA-peroxidase, lipase, bioactive compounds, cells, and drug molecules [22,23,24]. It has shown excellent promise to immobilize enzymes and develop hydrogels with unique functional moieties, which interact with biomacromolecules [25]. Furthermore, this is resilient to intestinal or gastric fluids comprising amylase and protease. Therefore, pectin-assisted hydrogel tablets and capsules with incorporated β-d-galactosidase might be beneficial to hydrolyze lactose in lactose-intolerant persons. Glycidyl methacrylate modified pectin has been commonly applied to synthesize water-insoluble hydrogels that appeared alternative support matrices for β-d-galactosidase immobilization because of elevated mechanical strength, and thus protect the enzyme molecule during hydrolytic reactions. Such hydrogels have also been used to immobilize urease [26,27,28]. Cargnin et al. [29] explored the potential of pinus residue/pectin-based bio-composite hydrogel matrices to immobilize β-d-galactosidase. The developed hydrogels comprising 0%, 5% and 10% of pinus residue showed enzyme immobilization abilities of 242.08, 181.27 and 182.71 mg enzyme/g dried hydrogel. In contrast to pinus residue, pectin-derived hydrogel matrix exhibited excellent capability for enzyme immobilization. The as-developed β-d-galactosidase-containing pectin hydrogels can be utilized in hydrolyzing lactose in lactose-intolerant people or dairy foods.

## 4. Hydrogels for Multienzyme Immobilization

It is observed that sometimes a single enzyme-mediated catalysis is not satisfactory. Superior efficiency of multienzyme-based reactions in living cells encourages the development of artificial multienzymatic biocatalytic systems [30], which can be applied to various applications, such as bioanalysis, diagnostic, bioproduct synthesis, bioremediation, and fuel cell development. Two or more enzymes that are capable of working together in a coupled or cascaded reaction are congregated to construct a multienzyme bio-system [31,32]. Developing co-immobilized multienzyme systems is an excellent substitute for classical multistep synthetic procedures, however, keeping multiple enzymes in a proper relative position on the support is the major challenge in the co-immobilization method [33]. In a recent study, two different biomaterials, including alginate and chitosan beads were utilized to co-immobilize pectinase, α-amylase, and protease. Optimal Na-alginate concentration was determined to be 2.5%, while the maximum enzyme loading ratio was 1:2:0.02 for protease, pectinase, and α-amylase, respectively. Likewise, the optimal conditions for multiple enzymes immobilization on glutaraldehyde-functionalized chitosan hydrogel beads were chitosan concentration [3%], glutaraldehyde [0.25%], activation (3 h) and cross-linking time (3 h). Based on these results, both these biopolymers could be used for designing multienzyme immobilized systems as a valuable and innovative technology for the remediation of polluted environments [34]. A glucose-sensitive hydrogel composed of N,N-dimethyl acrylamide (DMAAm), polymerizable sulfadimethoxine monomer, and sucrose particles was synthesized for covalent conjugation of catalase and glucose oxidase. The pH inside the hydrogel matrix fluctuates from pH 7.4 to 7.2 in a glucose level of 0–300 mg/dL in buffered-saline solution [35]. Table 1 summarizes the use of hydrogels for enzyme immobilization, improved attributes, and proposed applications [36,37,38,39,40,41,42,43,44,45,46,47].

## 5. Hydrogel-Based Sensors for Biomedical Applications

### 5.1. Cancer Monitoring

The detection and monitoring of cancer is among the major challenge for smart identification in the biomedical field. For early detection of cancer, considerable attention has been directed to altered expression of various biological molecules (i.e., glycans and proteins), which are related to cancer metastasis. The fabrication of novel biosensors capable of combining with good selectivity and sensitivity could be a valuable tool in diagnostic applications. Hydrogels-based biosensors and devices can be efficiently applied to such biomedical applications. Crulhas et al. [48] prepared highly selective and sensitive hydrogel microstructures to detect superoxide anions released by tumor cells. They developed a new electrochemical biosensor based on superoxide dismutase coupled ferrocene within a poly[ethylene glycol] diacrylate three-dimensional hydrogel network and characterized by cyclic voltammetry and electrochemical impedance spectroscopy. The newly developed device showed a broad linear operational range and a low detection limit, and excellent sensitivity. Due to high stability, selectivity, and reproducibility, this system was successfully applied to monitor inflammatory by-products, which were secreted by prostate tumor cells.

**Figure 1:**
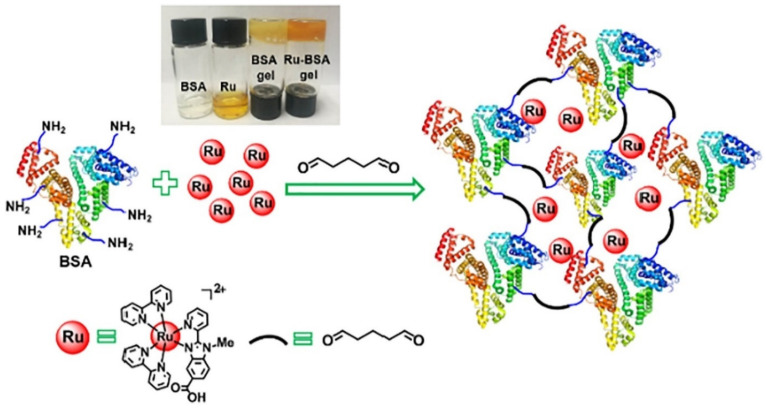
The schematic diagram of the Ru-BSA hydrogel. Reprinted from Reference [49] with permission from Elsevier. License Number: 5156040156264.

Zhao et al. [49] explored a new class of biodegradable albumin-ruthenium hydrogel that presents robust luminescence and high selectivity and thus widely applicable to cancer imaging and therapeutics. To synthesize this hydrogel, ruthenium complex and bovine serum albumin were simply dissolved in water followed by the inclusion of glutaraldehyde as a cross-linking agent (Figure 1). The resultant product was dialyzed to eliminate the redundant glutaraldehyde molecule and unbound ruthenium complex. The hydrogel was able to degrade in lysosome, and the ruthenium complex was separated from the Ru-BSA hydrogel matrix and rapidly accumulated in the mitochondria. Remarkably, inclusion of ruthenium complex into BSA hydrogel increased its cytotoxicity against Hep-G2 liver tumor cell lines and the selectivity [~6.8-fold] between cancer and normal cells. Hence, this approach offers a solid foundation in developing metal-based drugs as novel candidates to treat cancer.

**Figure 2:**
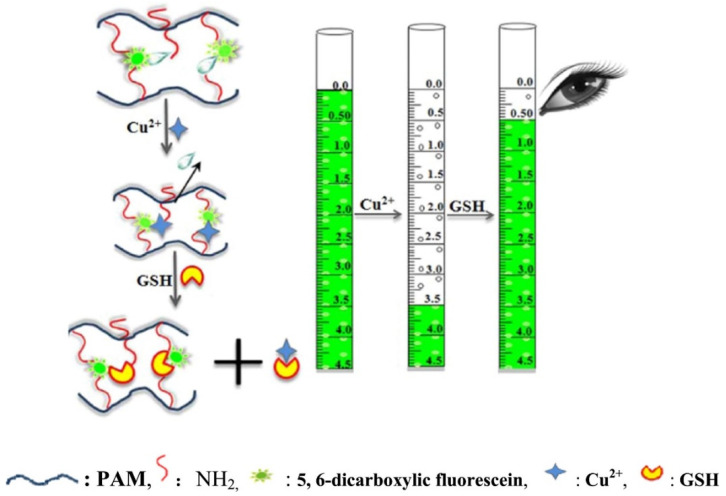
Hydrogel for volumetric response to glutathione (GSH). Reprinted from Reference [50] with permission from Elsevier. License Number: 5156040364062.

Another hydrogel-based sensor was introduced by [50] for visual sensing of glutathione, which possesses a critical role in numerous physiological functions, such as nutrient metabolism, antioxidant defense system (Figure 2). An imbalance in its concentration led to various diseases, including cancer, Parkinson, Alzheimer, and heart attack [51,52]. Glutathione is an imperative peptide that denotes an endogenous signaling molecule in tumors. The sensing system was produced using 5,6-bicarboxylic fluorescein crosslinked ammoniated polyacrylamide by sequential standardization of hydrogel particle size, buffer solution and swelling time. It was possible to analyze the hydrogel volume change using the biosensor by interpreting the graduation on a pipette-like thermometer by bare eye. The glutathione was found to depend on the volume in a specific range as the signal. Reasonable agreements between the as-developed biosensor and HPLC results for atuomolan tablet assays demonstrated the suitability of thermometer-type sensors to analyze real samples. In conclusion, the outcomes evinced the effectiveness of stimulus-responsive smart hydrogel and the applicability of thermometer-style visual biosensor design for real-time quantitation.

### 5.2. Detection of Cell Metabolites

**Figure 3:**
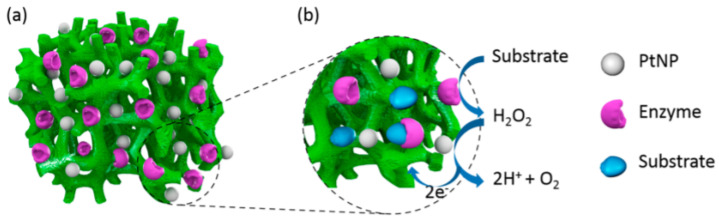
Schematic illustration of the general sensing mechanism of our CPH-based electrode platform. (**a**) PtNPs and enzymes were loaded onto hierarchically three-dimensional (3D)-porous PAni hydrogel matrices to form PAni hydrogel/PtNPs hybrid electrodes. (**b**) The PtNP-catalyzed sensing process of the biosensor based on PAni/PtNPs/enzyme hybrid films. Reprinted from Reference [55] with permission from the American Chemical Society.

Traditional methods for the detection of cell metabolites, such as cultivating cells and collection of intermittent cell or media culture display several drawbacks [53]. They necessitate large amounts of cells, reagents, and expensive operative processes. In addition, they are unlikely to furnish comprehensive information regarding the time evolution and dynamics of the examined system. To overcome these inadequacies, smart biological mechanisms-based strategies have been established for monitoring biosystems alterations in cells. In this context, hydrogel-based sensors play a noteworthy role in such bio-applications for detecting cell metabolites. [54] designed enzyme-based optical biosensor to detect H_2_O_2_ released by stimulated macrophages. The resultant sensing biosystem comprised of enzyme-harboring hydrogel structures and PEG hydrogel micropatterns were used to control cell sequestration and detection of the secreted cellular metabolites. The sensing system was constructed by entrapping horseradish peroxidase (HRP) and amplex red in the hydrogel matrix, where the Amplex Red can become fluorescent in the presence of HRP and H_2_O_2_. During analysis, the emergence of fluorescence in the hydrogel microstructures substantiates the effectiveness of the sensing system. The fluorescence intensity was found to proportional to be the concentration of analyte. The sensing elements coupled novel cell culture system could be upgraded by integrating additional biorecognition elements to facilitate the recognition of multi-metabolite at the cell site. To further corroborate this kind of technology, a versatile and scalable biosensor was fabricated by [55] to sensing human metabolites. This biosensing system consisted of a Pt nanoparticle-functionalized conductive polymer hydrogel (CPH) electrode (Figure 3). After characterization and calibration, the amperometric responses of the newly-developed system was used for detecting metabolites. This system presented outstanding biosensing performance for a large number of molecules, i.e., cholesterol, uric acid, and triglycerides in a wide linear range (uric acid, 0.07–1 mM; cholesterol, 0.3–9 mM, and triglycerides, 0.2–5 mM) with quick response time (∼3 s) and high sensitivity. In conclusion, the hydrogels-based biosensor shows good monitoring results with excellent specificity and sensitivity, however, great research efforts are being devoted to expanding their formulation and synthesis. Currently, the major hindrance in the use of hydrogel-based biosensing systems is their lack of stability over longer duration that need to be address for clinical diagnostics, healthcare monitoring, and biomedical devices.

### 5.3. Tissue Engineering

Tissue engineering is an imminent biomedical practice that integrates the utilization of cells, engineering, and advanced materials for improving or replacing a tissue scaffold to form new tissues. In this field, hydrogels have gained particular interest because of their capacity as immobilizing scaffolds for bio-receptors to measure and detect the level of desired biomolecules, kinetics, and interactions, which are essential for tissue engineering [56,57,58]. For example, [59] developed a new kind of optical adenosine triphosphate (ATP) biosensor for in-situ measurement of ATP in biological samples and tissues. As a multifunctional biomolecule, ATP plays a pivotal role in cellular metabolism due to its extracellular signaling activities. For the sensor development, two layers of sol-gel coating were applied to optical fiber probe end. The first layer consists of ruthenium complex to detect oxygen level changes determined by the oxidation of ATP by glycerol 3-phosphate and glycerol kinase enzyme encapsulated in the second layer. The as-fabricated platform appeared exceptional sensing tool and appropriate for the measurement of extracellular ATP contents in biological tissues.

**Figure 4:**
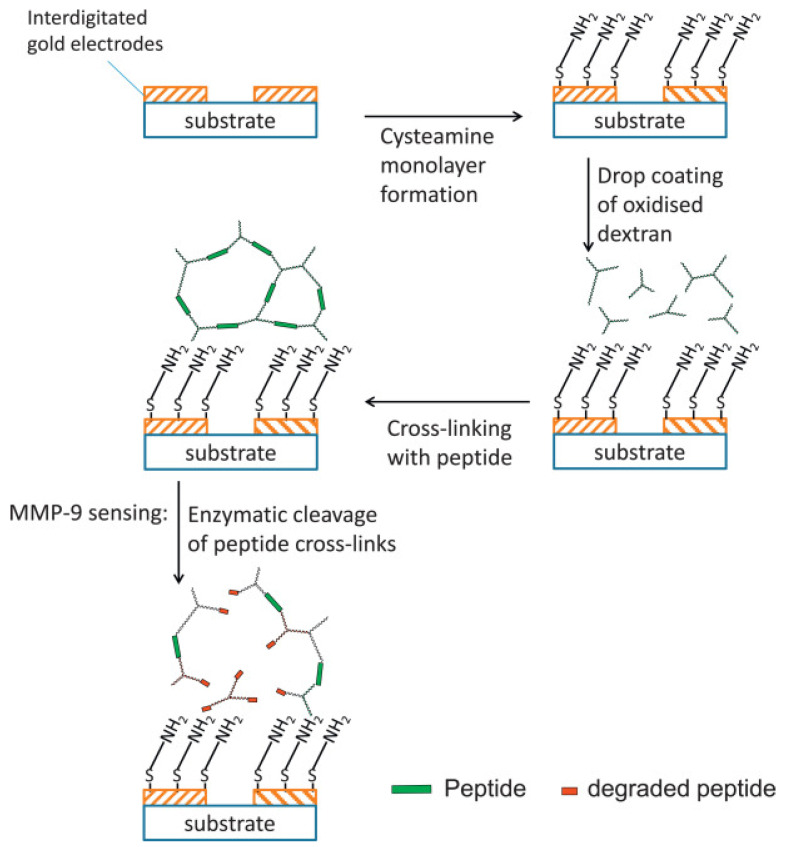
Schematic of sensor fabrication and the sensing process. Reprinted from Reference [61] with permission from Elsevier. License Number: 5156040634380.

Identification and monitoring of cellular activities is a crucial aspect in tissue engineering [60]. Cell proliferation, cytokine release, migration, and the secretion of specific molecules are valuable indicators that can be sensed to explore the scaffolds efficiency. Hydrogels-based biosensing devices are highly promising for this purpose. The activity determination of functional protein molecules is considered a fundamental aspect in tissue engineering applications. For instance, the detection of cells secreted matrix metalloproteinase (MMP) can be exploited as a potential biomarker. Biela et al. [61] proposed a disposable sensor for rapid and straightforward identification of MMP-9. This enzyme is a peripheral neuroinflammation biomarker in the chronic autoimmune disorder of the CNS, i.e., multiple sclerosis. It is likely to exert a paramount contribution in pathological as well as physiological processes. Currently, highly expensive magnetic resonance imaging (MRI)-based investigations are used for monitoring the subclinical disease activities in multiple sclerosis. In comparison to expensive MRI scans, the use of a disposable low-cost sensing system could be an alternative for MMP-9 detection. For this biosensors preparation, electrodes were coated with oxidized dextran following cross-linking of the system with peptides that contain explicit cleavage regions for MMP-9 (Figure 4).

A rapid sensor response was observed with a substantial impedance change within 5 min following the inclusion of MMP-9. Results revealed a successful identification of MMP-9 in a clinically pertinent range from 50 to 400 ng/mL. The sensor materials were generic and thus can easily be implemented for other proteases by assorting peptide cross-linkers with appropriate cleavage sites. Another interesting hydrogel system was reported by [62]. They used cyclic peptide-based progelator system that can assemble to form hydrogel in response to enzymes (MMP-2/9 and elastase). This system is useful for injection due to low viscosity as compared to gel systems. This hydrogel system could be used for minimum invasive delivery for therapeutic peptides for diseases with overexpression of MMPs or act as an injectable biomaterial for tissue engineering. Liang et al. [63] demonstrated the suitability of using gelatin containing hyaluronic acid hydrogel biocomposite in regenerative medicine as tissue-mimicking platforms to improve the survival of stem cells. They showed the perspective of chemical exchange saturation transfer MRI as a non-invasive label-free imaging approach for in vivo monitoring of dynamic changes in scaffold composition. After injecting hydrogel into immunodeficient rag2*^−/−^* mice brain and chemical exchange saturation transfer (CEST) MR images were captured at day 1 and 7 post-transplantation. A substantial reduction in chemical exchange saturation transfer signal was experimented in vivo at 1-week post-implantation. This label-free imaging technique could be helpful to develop various hydrogel preparations with augmented stability and diverse composition.

### 5.4. Wound Healing Monitoring

Wound healing monitoring is another fascinating application of hydrogels in biomedical field. Wound healing is an important physiological process that enables the replacement of devastated tissues in living entities. Given tunable features and properties, smart hydrogels have attracted incredible consideration as prodigious structures to stimulate immune cells for swift wound curing. Over the past few years, many research studies have emphasized the prospect of hydrogels utilization as promoters and monitoring devices for wound healing. Hydrogels are likely to play a key role in treating bacteria propagation or infections in the wound since their timely detection is important to prevent pain and associated complications.

**Figure 5:**
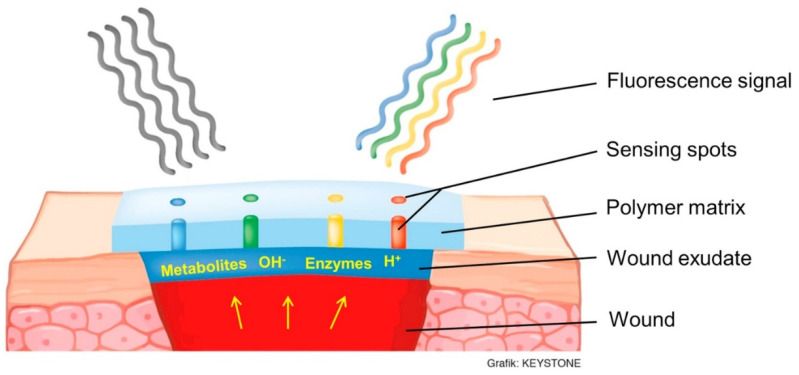
Scheme of the sensing principle for non-invasive wound monitoring based on the detection of pH-values and glucose concentrations. Reprinted from Reference [65] with permission from Elsevier. License Number: 5176700564947.

The level of pH in a chronic wound bed is a crucial parameter to assess the healing progress. Commercially available glass microelectrodes are not suitable for measuring the wound pH because of fragility and incapability of concurrently mapping multiple wound regions. To overcome this drawback and wound monitoring, Rahimi et al. [64] developed a low-cost pH sensor comprising two-screen printed electrodes and a conductive-selective polymeric membrane. Characterization analysis revealed that pH sensors showed an average sensitivity of −50 mV/pH and a linear relationship between the pH (range 4 to 10) and output voltage. Its biocompatibility was verified by exposing to human kertinocyte cell lines. Jankowska et al. [65] demonstrated a fluorescent detection platform for assessing the wound status and early stage distinguishing between an autonomously healing and chronic wound. The two most relevant fluctuating wound parameters, pH, and glucose concentration were monitored by this system. Carboxynaphthofluorescein (a fluorescent pH indicator dye), and HRP and glucose oxidase (GO)-based metabolite-sensing enzymatic system were conjugated on polysaccharide matrix for developing a functional hydrogel coating to monitor wound progress (Figure 5) [65]. The variations in enzyme concentration and metabolite in non-natural wound extract were transformed into a fluorescent signal.

In a recent study, thermosensitive hydrogel membranes were designed for enhanced wound curing using a novel amalgamation of thermoresponsive polymer (F-127), sodium alginate (biopolymer) and polyvinyl alcohol (PVA, synthetic polymer). The as-developed hydrogel membranes were hypothesized to meet all desired prerequisites for wound healing by exploitation unique features of conjugated polymers. They also control drug release at the wound region, which in turn increase the local concentration of drug. Characterization results demonstrated that the developed hydrogel membranes presented good and tensile strength and mechanical attributes to endure external frictional pressure, excellent swelling activities, and surface permeability for continuous release of conjugated drug molecules. A meaningfully greater zone of inhibition was recorded against *P.* *aregnosa* and *S. aureus* by amikacin-loaded hydrogel membranes. More importantly, tested animal models displayed considerably higher wound curative efficiency of hydrogel membranes regarding faster wound healing, better re-epithelization, and granulation tissue formation than that to negative and positive control groups. In conclusion, widespread in vivo and in vitro assessments evidently demonstrated a prospective wound dressing applicability of alginate-assisted hydrogel membrane and thus could be robust alternative wound healer for acute and chronic wounds [66].

### 5.5. Wrist Pulse and Cardiac Rhythm Monitoring

**Figure 6:**
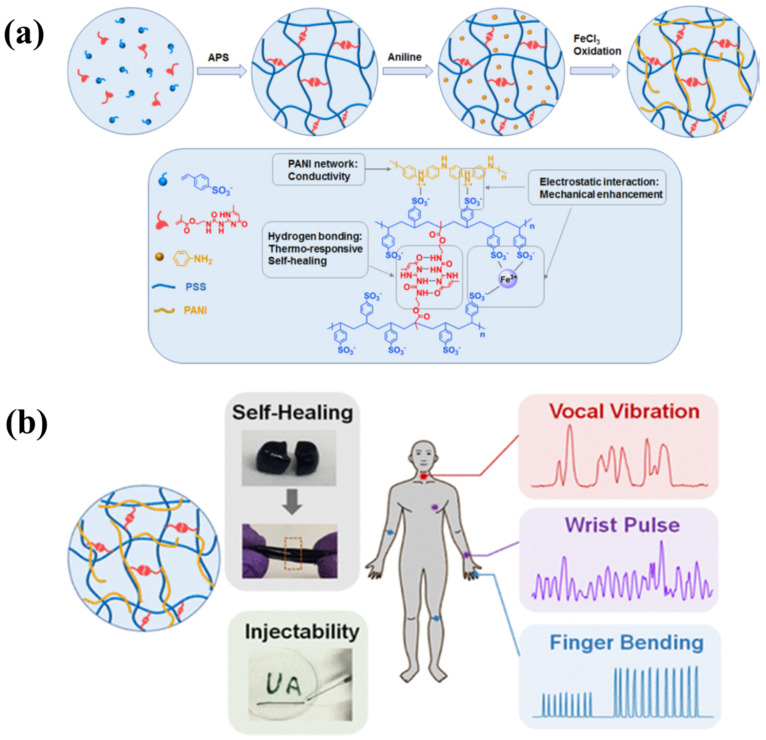
(**a**) Schematic illustration of synthesis process of the supramolecular conductive PANI/PSS−UPy hydrogels and the formation mechanism, (**b**) application in various human motions. Reprinted from Reference [68] with permission from the American Chemical Society.

In pre-clinical practice, monitoring artery pulse is an eloquent indicator for numerus aspects of the cardiovascular system, such as cardiac rhythm or arterial blood pressure. It is thought to deliver valuable information related to the non-invasive diagnosis of cardiovascular disorders. Hydrogel-based sensors with unique peculiarities have emerged as smart, flexible, and highly pertinent systems for such applications. They are capable of closely fitting the curvature and surface of human skins and can sense the pulse waves associated with cardiac rhythm, blood pressure, and wrist pulse with great precision and sensitivity. A large number of hydrogels formulations have been applied in wearable devices to monitor muscle movements, slight wrist pulse and joint motions [67]. Chen et al. [68] constructed a new conductive polymer hydrogel, in which multiple hydrogen-bonding 2-ureido-4[1H]-pyrimidinone (UPy) groups (as cross-linking points) were integrated into a fragile polyaniline/poly[4-styrenesulfonate] (PANI/PSS) network to couple high conductivity with outstanding injectability, stretchability, and self-healing ability (Figure 6). The PANI/PSS network development offers the hydrogel ionic transport-mediated electronic conduction, a linear response to external strain, and conductivity of 13 S/m accompanied by reliable and accurate monitoring of various human movements such as pulse beating and finger bending. Taking the advantages of reversible noncovalent cross-linkages, the hydrogels could be easily molded into various shapes and exhibit a complete self-healing within the shortest duration of only 30 s following injury. Combining the advent of supramolecular chemistry tools with conducting polymers allows the inclusion of multi functionalities in the hydrogel network and thus offer innovative frontiers in designing state-of-the-art biomaterials for applications in flexible electronics, wearable devices, and 3D printing.

In another investigation, biocompatible conductive hydrogel was fabricated by interpenetrating poly(-acrylamide-co-hydroxyethyl methyl acrylate) and polyaniline networks [69]. Owing to inherent coordination between the flexible P[AAm-co-HEMA] and conductive PANI network, the as-fabricated system showed high stability, excellent sensitivity, and durability to cyclic loadings. Conductive hydrogels-based strain sensing devices showed precise recognition of subtle vibrations, and recurrent large strains, including the activities of various human voiceprints, pulses, and joints. In addition, a prototype 2-dimenisonal (2D) sensor array was also prepared for sensing pressures or strains in two dimensions, which is useful for implantable sensors, wearable devices, touchpads, electronic skin, biomedical implants, and human-machine interfaces. Multifunctional strain sensors have captivated much interest in recent years for use in soft wearable devices. Nevertheless, it remains a great challenge to achieve a set of appreciable features, such as easy synthesis, high sensitivity, and superior mechanical properties. Hou et al. [70] fabricated high performance ionic strain sensors using agar/NaCl/polyacrylamide hydrogels for use in wearable devices. Pre-gel formulation can be injected into diverse shapes owing to thermo-reversible sol-gel attributes of agar. The newly fabricated hydrogels presented superior strain sensitivity and many mechanical features. The multifunctional ionic sensing device can monitor various human motions, i.e., slight wrist pulse, joint motions, and throat muscle movements.

The same research group designed another conductive, transparent, self-adhesive, and stretchable hydrogel by incorporating polydopamine-doped polypyrrole (PDA-PPy) nanofibrils into polyacrylamide (PAM) polymeric network [71]. Thanks to inner unique architecture and a conductive path created by the nanofibrils, the nanosystem displayed a high conductivity, and thus is widely applicable in sensing applications. The recoverable mechanical properties, stability, and great flexibility constitute hydrogel as ideal matrix for adhering to human skin and can be applied to monitor cardiac rhythm. Zhang et al. [72] devised a probable solution to prevent fluctuations and improve the sensitivity of sensing devices. They fabricated a composite hydrogel by integrating MXene (Ti3C2Tx), an interesting class of 2D metal carbides/nitrides/carbonitrides with high electrical conductivity and hydrophilic surfaces. MXene was combined with PVA hydrogel, characterized and the resultant system presented excellent tensile strain sensitivity, which is substantially greater than other similar hydrogel-based biosensors. The exclusive sensing ability could be extended to other applications involving touch sensing, 3D motions, and biosignal monitoring. Given excellent conformability, adhesiveness, and stretchability, MXene-based hydrogel can be considered a versatile material for artificial skin, soft robotics, and wearable electronics. Overall, the facile fabrication approach promises a broad variety of hydrogel-based composites as sensing materials.

### 5.6. Enzyme-Responsive Hydrogels for Drug Delivery

Enzyme responsive hydrogels were also exploited for drug delivery applications. Due to ease of injectability, they offer minimal invasive opportunity. Higher enzyme concentration at disease tissue can be targeted for such hydrogels. For instance, matrix metalloproteinases (MMP) were found higher in tumor cells. In this regard, MMP responsive systems are of quite importance for targeted drug delivery systems. Recently, Zhao et al. [73] used Temozolomide (TMZ) and O^6^-benzylamine (BZ) loaded MMP responsive hydrogel for treating TMZ resistant glioma and prevention of recurrence after surgical removal. In nude mice tumor model both in situ and subcutaneous, MMP responsive gel showed superiority over direct TMZ treatment as evidenced by prolonged survival and tumor growth inhibition [73]. In another report, proteolytic enzymes sensitive hydrogel for delivery of gemcitabine for pancreatic cancer treatment [74]. Poly-lysine-based hydrogel was found to control drug release through degradation by proteases. In xenograft tumor bearing mice, treatment with peritumoral injection of hydrogel (containing gemcitabine) and peritumoral gemcitabine showed similar survival rates. However, autopsies showed adherence of gel to tumor site which is helpful for local drug delivery and enzymatic degradation of gel avoid surgical removal [74]. Hydrogel microparticles responsive to MMP can also be used for pulmonary drug delivery to treat lung cancers [75]. Oral route of drug administration is preferable over other routes of administration due to several benefits such as ease of patient compliance and cost-effectiveness. Enzyme responsive hydrogel for drug delivery were also employed via oral route for treatment of colon cancer. Ma et al. [76] prepared crosslinked hydrogel for colonic specific release of 5-flurouracil. The release dependent upon colonic pH and enzymes as rat colonic fluid showed higher degradation of gel and faster drug release.

In addition to tumor treatment, applications of such enzyme responsive hydrogels have also been studied in other diseases as well. For example, triglycerol monostearate-based hydrogel loaded with triamcinolone acetonide for treatment of inflammatory arthritis [5]. The hydrogel showed flare-associated enzyme-like MMP responsiveness drug release at joint site. Significant higher release of drug was observed from hydrogel in presence of Esterase, MMP-2, MMP-3 and MMP-9. Furthermore, in vivo efficacy was also confirmed in murine K/BxN serum-transfer model of inflammatory arthritis [77]. In another report, proteolytic enzymes (overexpressed in inflammation) responsive hydrogel was employed to deliver tacrolimus [an immunosuppressive drug] to prevent immune response. In rats, this hydrogel was found to protect vascular allograft rejection for more than 100 days [78]. Furthermore, enzymes can be incorporated into hydrogel to form a substrate sensitive drug delivery system. For instance, glucose oxidase was incorporated into self-assembled peptide hydrogel to get glucose responsiveness. Insulin was loaded as drug and hydrogel showed glucose responsive insulin release [79]. This injectable hydrogel can act as smart insulin depot for diabetic patients. Antibodies responsive composite hydrogels for biopharmaceutical release were also been reported which is approach for smart delivery of next generation medicines [80].

### 5.7. Enzymatic Cross Linkable Hydrogels in Bioprinting

Bioprinting is advanced additive manufacturing technique allowing printing of cells along with hydrogel for tissue engineering. In this regard, a cell compatible crosslinking approach is required to give mechanical strength to hydrogel. Enzymatic crosslinking has benefit due to mildness of the reaction [81]. Zhou et al. [82] used enzymatic crosslinking of gelatin methacryloyl (GelMA) to improve rheological properties for bioprinting. Microbial transglutaminase was used for isopeptide bond formation, which improved the mechanical properties of gel for printing and secondary photo-crosslinking further stabilized the gel. Zhou et al. [83] also used microbial transglutaminase for GelMA bioprinting. In another report, Petta et al. [84] used the similar technique of dual crosslinking for hyaluronic acid-tyramine based hydrogel. They first used horseradish peroxidase and hydrogen peroxide for enzymatic crosslinking to produce bioink with proper extrudability and incorporated Eosin Y for photo-crosslinking. Bioprinted structures showed long-term stability and cell viability up to 14 days. Silk is a protein biopolymer and is widely used for tissue engineering. Silk/gelatin-tyramine based hydrogels was prepared with enzymatic cross-linking using horseradish peroxidase and hydrogen peroxide. They produced hydrogels with tunable properties by varying different components in order to get desired silk hydrogels for bioprinting. This enzyme sensitive behavior of hydrogel can be further extended to other biocompatible materials by introduction of enzymatic cross linkable groups in the structures.

## 6. Conclusions

Hydrogels are promising systems for the encapsulation of enzymes for various applications, especially biomedical applications owing to their biocompatibility. In the present review, we have discussed a potential application of hydrogels loaded with enzymes or hydrogels with capability to respond to particular enzymes. For biocatalysis, hydrogels offer superior enzyme loading capacity, stability, activity and shelf-life. Easy diffusion of several compounds through the hydrogel network is another auspicious property that is helpful for sensing applications. In this regard, a number of hydrogel systems have been utilized for biosensing for different biomedical applications such as tissue engineering, disease monitoring, wound healing, etc. In addition to enzyme encapsulation in hydrogels, enzyme responsive hydrogels are also potentially important in the biomedical field. They can be used for enzyme-responsive drug delivery systems and as bioinks.

Considering the limitations of hydrogels utilization, shelf-life, storage, and handling are common limitations. Hydrogels have a considerable amount of water and tend to dry over time if not stored in environmental conditions. For biosensing applications, compatibility/adaptation with a transducer is also required. For enzyme-responsive drug delivery applications, hydrogels should be specific in release action; however, a limitation of non-specific and premature release may hinder the applicability for tumor targeting. Additionally, hydrogels have to pass the regulatory requirements for biomedical applications, which require biocompatibility of the materials. Another hindrance is the cost and complexity of scale-up production as several hydrogels have complex synthetic routes. However, natural-sourced hydrogels are beneficial in this regard. For bioprinting applications, cross-linking should be done at biocompatible concentrations of enzymes in order to prevent cellular damage. At low concentrations, enzymes may be safe enough for cells but may provide insufficient mechanical properties to bioprinted constructs.

From future perspectives, the applications of enzyme-encapsulated and enzyme-responsive hydrogel may be extended and optimized using novel matrix systems with improved properties. For biocatalysis, significant efforts are dedicated to making robust immobilized enzymes systems enabling them to work in rough environmental conditions. In this regard, hydrogels may be further improved in future to meet such rigorous requirements. For biosensing, multi-targeted biosensing hydrogels may be designed to provide a cost-effective single detection kit to sense different targets. Additionally, improvement in lifetime, storage and time required for biosensing may need to improve for clinical translation of these systems. Three-dimensional printability of these hydrogels may be optimized for construction devices for biomedical applications. For this purpose, cross-linking for higher mechanical properties is a potential research area. Such 3D printed devices may be implanted in the body for biomedical applications.

## Figures and Tables

**Table 1:** Use of hydrogels for enzyme immobilization, improved attributes, and proposed applications.

Hydrogel	Enzyme	Improve Features	Proposed Application	Reference
Poly [vinylimidazole]/clay hydrogel	Alkaline protease	Retention of over 60% activity after 16 reuse cycles Enduring 35% remaining activity after 6th cycle	Biocatalysis	[36]
Carboxymethyl cellulose-based hydrogel	Cellulase	High storage and thermal stability Superb activity for rice straw hydrolysis than free enzyme In contrast to free enzyme, immobilization increased the rice straw hydrolysis percentage by 74.75% after 1 h and 42.30% after 48 h.	Enzymatic degradation of lignocellulosic biomass	[37]
Zwitterionic poly[carboxybetaine] microgels	Chymotrypsin	Enhanced enzymatic stability Outstanding reusability retaining 72% of activity after 10 cycles Increased shelf-life Superior activity at high pH	Enzyme immobilization for extended stability and activity	[38]
Pectin-based hydrogels	β-d-galactosidase	Excellent support for enzyme immobilizationHigh catalytic capacity	Hydrolysis of lactose	[39]
Graphene oxide@CMC-g-poly[AMPS-co-AAm] hydrogel	Cellulase	Retaining 60% bioactivity at 90 °C Enhanced storage stability and specific activity 154.8% improved bioconversion of alkali-treated sugar beet pulp	Enzymatic hydrolysis of lignocellulosic biomass	[40]
Methacrylate substituted polyphosphazene	Lipase entrapment	High enzyme loading Over 65% retention of enzyme activity	Enzyme immobilization for imprved stabiltiy ansd reusuability	[41]
Molecular hydrogelator and sodium alginate	Lactase	Good mechanical strength Recyclability Excellent preservation of enzyme activities	Enzyme immobilization for biological applications	[42]
Zinc-based hydrogels	Glucose oxidase and horseradish peroxidase	Remarkably enhanced operational stability High enzyme activity	Catalysis of Knoevenagel reaction and enzyme immobilization	[43]
Poly[ethylene glycol] [PEG]-based hydrogels	Glucose oxidase	Higher enzymatic activity Activity retention for one week without leakage	Enzyme immobilization for improved stability	[44]
Polymerized ionic liquids-based hydrogels	*Candida antarctica* lipase B	Increased enzymatic activity than free enzyme Elevated enantiomeric excess and no leakage of active enzyme Easily recovery and reusability	Development of reusable and active and biocatalysts	[45]
Poly[ethylene glycol][PEG]-based interpenetrating polymeric network (IPN) hydrogels	Glucose oxidase	Maintenance of 80% enzyme for one week	Enzyme immobilization for catalysis	[46]
Thermo-responsive hydrogels	Pepsin	Preservation of catalytic sites at high temperatures Activity recovery and reuse for 10 continuous cycles Catalysis of complex reaction batches at elevated temperatures without activity loss	Catalytic applictaions	[47]
